# Nutritional Value of Meat and Meat Products and Their Role in Human Health

**DOI:** 10.3390/nu16101446

**Published:** 2024-05-11

**Authors:** Joanna Stadnik

**Affiliations:** Department of Animal Food Technology, Faculty of Food Science and Biotechnology, University of Life Sciences in Lublin, Skromna 8, 20-704 Lublin, Poland; joanna.stadnik@up.lublin.pl

Meat and meat products are among the most nutrient-dense food sources in the human diet. They fulfill most of our bodily requirements, acting as important sources of energy and a variety of essential nutrients, such as high-quality protein, several micronutrients (such as readily bioavailable iron, zinc, and selenium), vitamins (B6, B12, and folic acid), and bioactive compounds (taurine, carnitine, carnosine, ubiquinone, glutathione, and creatine) which are needed for a whole series of metabolic functions. Meat and meat products played a vital role in human evolution and are important components of a healthy and well-balanced diet [1,2,3]. The nutritional composition of meat and meat products differs depending on the cut, leanness, and processing level of the meat. Meat and meat products may also have other nutritive and non-nutritive components, such as sodium, saturated fat, nitrites, heterocyclic aromatic amines, and polycyclic aromatic hydrocarbons, which are associated with negative health-related outcomes. For this reason, frequent and excessive meat consumption, especially of red and processed meat (e.g., meat that is grilled, cured, or smoked), is currently a topic of scientific controversy, and there is a great deal of confusion as to the relationship between such consumption and adverse health outcomes, such as increased risk of cardiovascular diseases and multiple types of cancers, particularly colorectal cancer [4,5,6,7,8]. Moreover, concerns have also been raised regarding the environmental and climate-related effects of animal-sourced food production [9,10]. However, recommendations to limit or eliminate meat from our diets to minimize its environmental impacts present a broad spectrum of nutritional consequences, which vary by nutrient status, population, life course phase, and replacement food [3].

These matters are addressed in the Special Issue “Nutritional Value of Meat and Meat Products and Their Role in Human Health”, which aims to present recent developments in the field of the nutritional value and health effects of meat and meat products.

Twenty-four manuscripts were submitted for consideration for this Special Issue of Nutrients, and all of them were subjected to the journal’s rigorous peer-review process. In total, thirteen papers (nine research papers, two reviews, and two other papers) were accepted for publication and inclusion in this Special Issue. The contributions are listed below:

Contribution 1 evaluated alpha-glucosidase-inhibiting peptides obtained from dry-cured pork loins inoculated with probiotic/potentially probiotic strains of LAB. The most promising sequences (VATPPPPPPPK, DIPPPPM, TPPPPPPG, and TPPPPPPPK) showed potential as antidiabetic agents.

Contribution 2 conducted a cross-sectional analytical study to assess the frequency of meat intake and willingness to reduce consumption on health and environmental grounds in residents of the Lisbon metropolitan region. The findings revealed that less frequent meat consumers were more amenable to reducing their meat consumption for environmental and health reasons than those who consumed meat more frequently.

Contribution 3 characterizes the consumption levels of total beef (i.e., any beef type) and individual beef types (fresh lean beef, ground beef, and processed beef) in Americans aged 2 years and older over an 18-year period using NHANES 2001–2018 data. The findings of the study indicate that beef consumption in children, adolescents, and adults declined, while consumption remained consistent in older adults. To date, no studies have evaluated beef-specific usual intake data on both a per capita and consumer basis.

The findings of this study were also mentioned in Contribution 13, in which the Authors emphasized the environmental effects of producing and consuming beef, and subsequent recommendations among expert bodies encouraged populations to reduce their red meat intake to support human and planetary health.

Contribution 4 investigated the relationship between pork intake (including fresh pork and processed pork) and overall nutrient intake, as well as the ability to meet nutrient recommendations in US children and adults, using the National Health and Nutrition Examination Survey (NHANES) 2011–2018. The research showed that pork intake was associated with higher intake and adequate amounts of certain key nutrients in children (age 2–18 years) and in adults (age 19+ years).

Contribution 5 assessed the potential unintended consequences of limiting meat and poultry by modeling the effect of removing a serving of meat/poultry on nutrient profiles of the Healthy Dietary Patterns (HDPs) identified in the Dietary Guidelines for Americans, 2020–2025, and assessed whether the modeled changes led to meaningful differences in intake. The results of this dietary modeling analysis show that removing a serving of meat or poultry could lead to decreases (10% or more from baseline) in protein and several key nutrients (iron, phosphorus, potassium, zinc, selenium, thiamine, riboflavin, niacin, vitamin B_6_, vitamin B_12_, and choline) as well as cholesterol and sodium in the HDPs.

Contribution 6 examined the association between Stroke Belt (eight southern U.S. states) residence and colorectal cancer incidence in relation to food consumption. This cross-sectional study found that Stroke Belt residency was associated with consuming greater quantities of meat, particularly red meat. The same associations were not observed for colorectal cancer quartiles and meat consumption, nor were there any associations between state residency and healthy food consumption.

Contribution 7 aimed to reveal the association between the intake of 23 nutrients and the risk of dementia in a large cohort after adjusting for a wide range of confounding factors. A greater risk of dementia was associated with no alcohol intake (compared with moderate to higher intake), higher intake of total sugars and carbohydrates (compared with lower intake), the highest or lowest fat intake (compared with moderate intake), quintiles of highest or lowest magnesium intake (compared with the quintile of the second highest intake), and the highest protein intake (compared with moderate intake).

Contribution 8 explored early feeding practices related to the introduction of beef in the rural US west (Idaho); parental perceptions of beef as a first food; and associations between early dietary beef, protein, iron, zinc, and choline intake and child cognition at 1–5 years of age. The findings of the study indicate that higher intake of beef, zinc, and choline at 6–12 months was associated with better attention and inhibitory control at 3–5 years of age. The results of this study show that early-life beef consumption patterns are related to cognitive outcomes.

Contribution 9 defined the state of the art of lipid nutritional indexing in poultry meat and, on this basis, conceived three progressive indexes (QuantiN-3 Index; Healthy Fatty Indexes 1 and 2) that more effectively explore potential uses in the determination of nutritional properties by comparing two divergent poultry genotypes with different growth rates and meat traits.

In Contribution 10, the authors conducted a narrative literature analysis on poultry consumption and human cardiometabolic health-related outcomes. Nutritional profiles of commonly consumed poultry products, consumption trends, and dietary recommendations in the United States were described. The associations between (and effects of) poultry consumption on body composition and body weight, type II diabetes mellitus, and cardiovascular disease were discussed.

Contribution 11 narratively reviewed the most recent literature on the relevance of micronutrient deficiency risk in vegetarian or vegan children based on the available data. It primarily focused on their intake of iron, zinc, iodine, and vitamins B12 and D. The findings revealed a lack of well-designed studies to assert the risk of micronutrient deficiency in vegetarian children. The results of the study indicate a need for education, regular medical/dietic supervision, and individually assessed supplementation.

Contribution 12 responds to Contribution 13, stating that sustainability was outside the scope of Contribution 3. This research endeavored to provide objective data on beef intake trends using a publicly available database (i.e., National Health and Nutrition Examination Survey (NHANES)) in the context of the example patterns from the 2020–2025 Dietary Guidelines for Americans (DGA).

Overall, this Special Issue, “Nutritional Value of Meat and Meat Products and Their Role in Human Health”, identifies several potential research opportunities and future directions:

- Comprehensive analysis of intake of individual meat types across all life stages is needed to evaluate the relationships between red and processed meat intake and disease risk and to support the development of evidence-based dietary advice.
- Public health interventions aimed at reducing diet-related health disparities should consider the confluence of location and meat consumption in the development of lifestyle behavior change strategies and targeting practices.
- Models are needed to help define what foods would need to be consumed in greater quantities to replace nutrients previously gained from consuming meat if it was removed from the diet.
- More data on the relationship between residing in a high stroke area, colorectal cancer incidence levels, and red meat and processed meat consumption are needed.
- The ways in which access to preventative medical care and healthcare professionals confounds poor dietary choices associated with chronic diseases should also be explored further.

In light of the success of this Special Issue, we are pleased to announce a second Special Issue on this topic, entitled “Nutritional Value of Meat and Meat Products and Their Role in Human Health-2nd Edition”. We are seeking original research papers and reviews on the nutritional value and health effects of meat and meat products. We believe that this Special Issue will broaden the horizons of our knowledge on the role of meat and meat products in human health.
